# Efficient Biosynthetic Fabrication of Spidroins with High Spinning Performance

**DOI:** 10.1002/advs.202400128

**Published:** 2024-03-23

**Authors:** Baoyang Lin, Jingjun Xie, Bingbing Gao, Bingfang He

**Affiliations:** ^1^ College of Biotechnology and Pharmaceutical Engineering School of Pharmaceutical Sciences Nanjing Tech University Nanjing 211816 China

**Keywords:** Amy‐6rep, electrospinning, nanogenerator, sequence modification, spidroin

## Abstract

The unique 3D structure of spider silk protein (spidroin) determines the excellent mechanical properties of spidroin fiber, but the difficulty of heterologous expression and poor spinning performance of recombinant spider silk protein limit its application. A high‐yield low‐molecular‐weight biomimetic spidroin (Amy‐6rep) is obtained by sequence modification, and its excellent spinning performance is verified by electrospinning it for use as a nanogenerator. Amy‐6rep increases the highly fibrogenic microcrystalline region in the core repeat region of natural spidroin with limited sequence length and replaces the polyalanine sequence with an amyloid polypeptide through structural similarity. Due to sequence modification, the expression of Amy‐6rep increased by ≈200%, and the self‐assembly performance of Amy‐6rep significantly increased. After electrospinning with Amy‐6rep, the nanofibers exhibit good tribopower generation capacity. In this paper, a biomimetic spidroin sequence design with high yield and good spinning performance is reported, and a strategy for electrospinning to produce an artificial nanogenerator is explored.

## Graphical abstract

A high yield and low molecular weight bionic spider silk protein (Amy‐6rep)is obtained by sequence modification and verifies its excellent spinning performance by electrospinning, which can be used as nano generator. The expression of Amy‐6rep is increased by about 200%, the self‐assembly performance is significantly improved, and the nanofibers showed good tribopower generation ability after electrospinning.

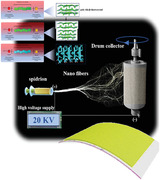

## 1 Introduction

Among the natural macroscopic materials, the ampulla dragline is one of the main materials with excellent biocompatibility and mechanical properties. As early as the ancient Greek period, there are records of people using spider silk to stop bleeding and heal wounds.^[^ ^1^ ^]^ Due to its performance of cell adhesion, spider silk has been applied to promote the viability and survival rate of cell culture in vitro.^[^ ^2^ ^]^ Since spider silk is composed of spider silk protein(spidroin), its potential functionalization of protein genes gives it a natural advantage in cell‐specific attachment.^[^ ^3^ ^]^ At the same time, spider silk has good water vapor permeability, making it closer to natural skin tissue, providing a more biomimetic physiological environment for cells, and therefore conducive to inducing tissue regeneration.^[^ ^4^ ^]^ Among the many methods of protein molding, fiber is one of the most widely concerned technologies.^[^ ^5^, ^6^ ^]^ Biomolecular fibers based on electrospinning can achieve cell‐directed migration and repair to promote wound healing,^[^ ^7^ ^]^ so electrospinning is an effective means to prepare spidroins as fibers. However, the peculiarity of spiders makes it difficult to obtain natural silk on a large scale, many efforts have been devoted to creating artificial spider silk, mainly by heterologous expression of recombinant spidroins, and then spinning the fibers in artificial spinning devices. However, the high GC content of the natural spidroin gene, the highly repetitive amino acid sequence in the repetitive core region, the high specific amino acid content and the high molecular weight pose a huge challenge to its efficient heterologous expression,^[^ ^4^ ^]^ which limits the wide application of spider silk and also limits the ability to prepare recombinant spider silk based on electrospinning technology. Therefore, strategies are urgently needed for more effective heterologous expression of major ampullate spidroin (MaSp).

Over the past two decades, many strategies have been developed to improve the heterologous expression efficiency of MaSp. One approach is to design a mini spidroin consisting of an end domain and a central repeat region that is greatly shortened compared to the natural spidroin template. This spidroin can be produced in bioreactor culture at high expression levels (>20 g L^−1^), which makes the process economically viable.^[^ ^8^ ^]^ Notably, the silk formation ability of the mini‐spidroins was significantly reduced at low concentrations (at least 50% w/v is required to form silk), which indirectly makes their high yield less attractive. To improve silk‐forming ability, the alanine motif in the central repeat region of MaSp was replaced by an amyloid polypeptide, and the number of central repeat regions of MaSp was increased to obtain ultrahigh‐molecular‐weight recombinant spidroin.^[^ ^9^ ^]^ The obtained insoluble aggregates were then solutized and spun with organic solvents. This method allows the expression of the giant spider protein to be spun into fibers with high tensile strength, but the protein yield falls far short of what is required for industrial production. By appropriately increasing the number of central repeat regions required for amino peptide replacement with mini‐spidroin, it is possible to achieve a relative balance between silk‐forming performance and heterologous expression efficiency and achieve volume production of high‐performance recombinant spidroins.

Here, we combined a low‐molecular‐weight spidroin strategy with a sequence modification strategy involving amyloid polypeptide replacement based on *β*‐folded crystal structure similarity and proposed an ideal number of microcrystalline regions, increasing the number of highly fibrogenic microcrystalline regions in the core repeat region of natural spidroins to 6 with limited sequence length. Finally, bionic spidroins with high yields and low molecular weights were obtained by constructing a heterologous expression vector for *Escherichia coli*. Owing to the low sequence repetition of the amyloid polypeptide in bionic spidroin, the heterologous expression yield of the bionic spidroin Amy‐6rep increased by ≈200%, which laid the foundation for subsequent mass spinning. A reasonable increase in the number of microcrystalline regions was proven to improve the self‐assembly performance of biomimetic spidroins but had no significant effect on the level of heterologous expression. Notably, we adopted a simple inclusion body washing method for bionic spidroin, expressed in precipitation form, and the results showed that the recovery rate of this purification method reached ≈80%, which significantly improved the recovery efficiency compared with that of the previous Ni‐NTA purification method and indirectly improved the production of bionic spidroin. After the bionic spidroin was dissolved in hexafluoroisopropanol (HFIP), we obtained spidroin fiber spun from a pure spidroin solution by electrospinning for the first time. By using a simple power generation device, we confirmed that the obtained nanospidroin fibers have good frictional power generation capacity. Moderate current stimulation has been shown to be beneficial for wound recovery,^[^ ^10^ ^]^ and the application of nanogenerators in medical applications and wound management has often been reported, but excellent self‐friction power generation performance requires the material to have good mechanical properties.^[^ ^11^, ^12^ ^]^ Although some wound dressings are made of biomaterials with high biocompatibility, they have poor tensile properties and can lead to secondary wounds in special cases, such as wounds on the wrist, knee, and other joints. While textiles can quickly stop bleeding, most existing textiles are made of harmful chemicals that can cause allergic reactions, secondary skin lesions, and infections and are difficult to degrade, causing damage to the environment. Because spider silk protein has good biocompatibility, biodegradability, nanogenerator performance and excellent mechanical properties, wound dressings based on spider silk protein nanogenerators have unique advantages in the fields of medical applications and wound management. These results indicate that Amy‐6rep, a low‐molecular‐weight bionic spider silk protein, has a wide range of potential applications in industrial mass production of spider silk proteins, medical applications, and wound management.

## 2 Results and Discussion

**Figure 1:**
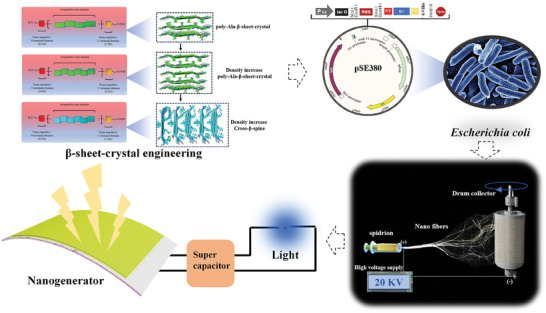
Biosynthesis and production of biomimetic nano spider silk made from low‐molecular‐weight biomimetic spidroin electrospinning for nano generation. Due to the limited sequence length, the polyAla motif of the natural spidroin MaSp1 was shortened, and the number of microcrystalline regions was increased to reduce the difficulty of heterologous expression. On this basis, the polyalanine feature motif was replaced with an amyloid polypeptide sequence with a cross‐*β*‐spine structure to further reduce the difficulty of expression and enhance the mechanical properties of the resulting protein. Biomimetic spidroin was biosynthesized from engineered bacteria and purified into a spinning paste. Biomimetic nano spider silk with triboelectric power generation performance was obtained by high‐voltage electrospinning, and a nanogenerator was prepared by using this property.

Generally, spidroins are composed of three parts: the more conserved N‐terminal (NT) nonrepeat region, the C‐terminal (CT) nonrepeat region, and the highly specific repeat core region. Recent studies have shown that N/CT is related to the self‐assembly of spidroins.^[^ ^13^, ^14^ ^]^ The repeat core region is mainly directly related to the spinning performance of spidroins, and the repeat core region shows great differences between different types of spider silk, resulting in significant differences in spinning performance.^[^ ^15^, ^16^, ^17^ ^]^ We mainly focused on major ampullate spider silk, which can have a tensile strength of 1–2 GPa and a strain at break of up to 30%, depending on the species.^[^ ^18^ ^]^ The major ampullate spider silk is composed mainly of major ampullate spidroins (MaSp), whose repeat regions are dominated by feature blocks of poly‐Ala residues interspersed with Gly‐rich repeat sequences. There can be up to 100 poly‐Ala repeats in a single MaSp, which are mainly stacked with 𝛽‐sheets to form nanoscale crystals, in which 𝛽‐strands from adjacent sheets are interrelated in a zip‐like structure.^[^ ^19^, ^20^ ^]^ Recently, a special main ampulla glandular filament was found; this filament is composed mainly of a low‐molecular‐weight protein with only 191 amino acids in the protein core sequence; thus, it was called MaSp1s (short). Studies have shown that the protein fibers formed by MaSp1s exhibit excellent mechanical properties, such as tensile strength.^[^ ^21^ ^]^ On the basis of these findings, through fiber energy analyses, we constructed a core sequence of the MaSp1 Rosetta energy (services.mbi.ucla.edu/zipperdb)^[^ ^22^ ^]^ and found that although its core sequence usually has repeated characteristics and a typical characteristic motif is lacking, its energy distribution is regular. MaSp1s contain eight microcrystalline regions with a high tendency to fuse within a limited sequence length (Figure S2, Supporting Information). Therefore, we believe that the microcrystalline region formed by the shorter polyalanine motif may increase the probability of collision between microcrystalline regions by increasing the density, thus promoting the formation of *β*‐folded microcrystalline structures and increasing the self‐assembly performance of proteins to obtain improved spinning performance. Moreover, previous studies have suggested that the size of nanocrystals in spider silk should be 6.5 nm to maximize the strength of silk fibers, as larger crystals can lead to uneven stress distribution in the material, leading to earlier fiber breakage. Combined with molecular dynamics simulations, studies have revealed that β‐nanocrystals limited to 3 × 4 × 7 nm have higher hardness, strength, and mechanical toughness than larger nanocrystals. Through nanoscale restrictions, modified nanocrystals can most effectively utilize the uniform shear deformation of hydrogen bonds combined with the emergence of dissipative molecular stick‐slip deformation, thus significantly improving their mechanical properties.^[^ ^23^ ^]^ There are 5–7 *β*‐strands corresponding to this size, and the number of amino acids contained in each *β*‐strand is 6–8. Therefore, we used NT‐4Rep‐CT (40.4 kDa), a mosaic minicarachnid protein already available in our laboratory, as the template (NT from MaSp1 of *Euprothenops. Australis*, E. australis , CT from MiSp of *Araneus. Ventricosus*, bracketing a short repetitive region from *E. australis*). When the sequence length was held constant, the number of polyalanine motifs decreased to 7, the number of polyalanine blocks increased to 6, and the bionic spidroin NT‐Cs‐6rep‐CT (39.8 kDa) was obtained with increased microcrystalline region density (**Figure** **1**a). In contrast to the *β*‐folded structure formed in the microcrystalline region of spidroin, amyloid proteins can further form a highly ordered cross‐*β*‐spine structure after the formation of a *β*‐folded structure.^[^ ^24^ ^]^ This structure is a network of noncovalent interactions between adjacent *β*‐chains through hydrogen bonding and adjacent *β*‐lamellar layers through electrostatic interactions, π–π accumulation, and hydrophobic effects. Amyloid nanofibers have extraordinary mechanical properties. We hypothesized that the amyloid protein is less difficult to synthesize than the characteristic polyalanine motif in spidroin due to its lower sequence repeatability. Therefore, based on the spidroin Cs‐6rep, we replaced the polyalanine motif in the microcrystalline region with an amyloid polypeptide. We selected an amyloid fragment, Fgailss (GRAVY:1.557), which is well known and has a grand average of hydropathicity similar to that of poly‐Ala (GRAVY: 1.8), in the amyloid database. Finally, the biomimetic spidroin NT‐Amy‐6rep‐CT (39.6 kDa) was obtained.

**Figure 2:**
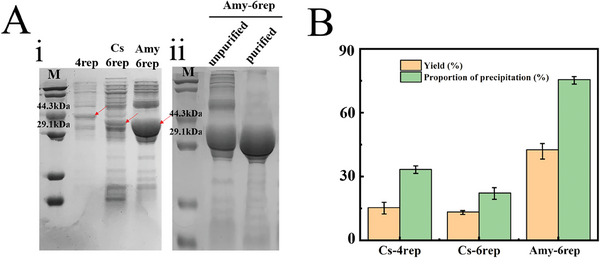
Characterization of the yield of biomimetic spidroins. A) SDS‒PAGE analysis of biomimetic spidroins. Note: M: protein marker; Line 1: 4rep (the primitive biomimetic spidroin, 40.4 kDa); Line 2: Cs‐6rep (the biomimetic spidroin with increased microcrystalline region density, 39.8 kDa); Line 3: Amy‐6rep (the biomimetic spidroin with increased microcrystalline region density and amyloid polypeptide replacement (39.6 kDa)) (i). B) Summary of the protein yield and proportion of precipitates for all biomimetic spidroins used in the study. The expression level was calculated as the percentage of overexpressed biomimetic spidroin relative to the total proteome of the corresponding *E. coli* strain, as measured via densitometric analysis of Coomassie blue‐stained SDS‒PAGE gels. Error bars represent standard deviations.

All the spidroins were constructed and expressed by optimized synthetic DNA expression vectors to reduce duplication of coding sequences. All the proteins were constructed and expressed by optimized synthetic DNA expression vectors to reduce duplication of coding sequences. The three chimeric spidroins were expressed on ≈15–50% of the host cells. Interestingly, all the mini‐chimeric spidroins in this study were expressed mainly in the form of inclusion bodies, while a small amount of soluble spidroin was expressed, which was different from the high solubility of mini‐spidroin NT‐2rep‐CT reported previously.^[^ ^25^ ^]^ We hypothesized that this difference may be caused by the increasing number of core regions of spidroins, which results in an increase in overall hydrophobicity and decreased solubility. Therefore, all proteins in this study were classified. The soluble forms of all the proteins were purified by Ni‐NTA affinity chromatography (Figure S6, Supporting Information) for subsequent characterization of the solution structure and self‐assembly performance. The inclusion body expression forms of all proteins were purified by inclusion body washing, and the lyophilized proteins were dissolved in HFIPsolution to prepare a spinning solution. Notably, the expression level of Amy‐6rep increased by ≈200% owing to the ease of sequence modification (Figure **2**A). Previous studies have shown that amyloid replacement can improve the heterologous expression of recombinant spidroin, but the effect is limited.^[^ ^9^ ^]^ Our experimental results showed that the application of the amyloid replacement strategy to mini‐spidroin significantly improved the efficiency of allogenic expression. Moreover, since all proteins in this study were expressed mainly in the form of inclusion bodies, we adopted a simple inclusion body washing method, and the results showed that the purification rate of this method reached ≈80%, and the productivity reached ≈535 mg purified per g dry cell (Table S1, Supporting Information). Compared with that of the previous Ni‐NTA purification method, the recovery efficiency of the proposed method was significantly improved, and the production of bionic spidroin indirectly increased. To demonstrate the potential for large‐scale production based on our low‐molecular‐weight amyloid bionic spider silk protein sequence modification, we performed high‐density fermentation of Amy‐6rep using a bioreactor. The curves of induction time and protein titer were obtained by using the relationship between yield and expression, and the maximum titer of the biomimetic spider silk protein Amy‐6rep was finally determined to be 12 ± 0.40 g L^−1^ (Figure S3, Supporting Information). The titer was higher than that of other recombinant spider silk proteins at present.^[^ ^8^, ^9^, ^26^ ^]^

**Figure 3:**
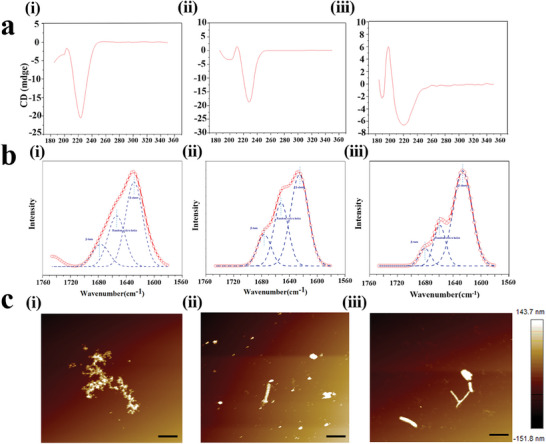
Microstructure and self‐assembly characterization of three biomimetic spidroins. a) Analysis of three biomimetic spidroins by circular dichroic chromatography. 4rep (i); Cs‐6rep (ii); Amy‐6rep (iii). b) Analysis of three biomimetic spidroins by Fourier transform infrared spectroscopy. 4rep (i); Cs‐6rep (ii); Amy‐6rep (iii). c) Analysis of three biomimetic spidroins by atomic force microscopy. 4rep (i); Cs‐6rep (ii); Amy‐6rep (iii).

The molecular structures of all spider proteins were characterized by circular dichroism (CD) spectroscopy. As shown in **Figure** **3**i, the CD spectra of 4REP and 6REP are similar, with a strong negative peak at ≈223 nm and a positive peak at ≈203 nm, indicating that the molecular conformation of the two proteins is mainly an α‐helical structure and parallel β‐sheet. In contrast, Amy‐6rep, an amyloid polypeptide‐replaced bionic spider protein, showed a significant positive peak shift at 199 nm and a wide negative peak near 220 nm, indicating that it mainly existed in a more stable antiparallel β‐sheet conformation. This finding suggested that the replacement of amyloid peptides may increase the stability of the *β*‐fold structure in the microcrystalline region of spidroins. In addition, a negative peak at 223 nm indicates partial retention of the alpha‐helical conformation of the protein. In Figure 3ii, the Fourier transform infrared spectroscopy (FTIR) spectrum (amide I band) was deconvoluted to analyze the secondary structures of all spider proteins. The absorption peaks at 1685, 1655, and 1628 cm^−1^ belong to the *β*‐turn, random coil/spiral, and *β*‐sheet conformations, respectively. Notably, the *β*‐sheet content of 4REP was 46.7%, whereas that of 6rep increased to 55.5%, indicating that an increase in the density of the microcrystalline region may increase the probability of collision between the microcrystalline regions, thus promoting the formation of *β*‐folding. The *β*‐sheet content of Amy‐6rep further increased to 65.2%, indicating that the replacement of the amyloid polypeptide may increase the tendency for *β*‐folding in the microcrystalline region of the spider protein. In particular, the CD and FTIR spectra were consistent with the AFM observations, and the self‐assembly performance of the modified spider proteins was significantly enhanced.

The repetitive domain in the recombinant spider protein sequence is thought to contribute to the formation of *β*‐sheets and promote protein self‐assembly.^[^ ^27^ ^]^ Therefore, we further investigated the self‐assembly of these recombinant spidroins by AFM. The morphology of the synthesized self‐assembled material is shown in Figure 3iii. Overall, the self‐assembly of the three chimeric spider proteins followed a process of heterogeneous nucleation, in which spider protein molecules first fold into nanoparticles, which then further assemble into larger aggregates. Preliminary nanoparticles appear in the early stages of protein self‐assembly. However, as shown in Figure 3ci, the self‐assembled 4REP spider protein produced well‐dispersed nanoparticles after incubating at 300 rpm for 36 h. The results showed that under our conditions, these 2REP spider proteins had difficulty further self‐assembling into dense aggregates. This is reasonable because the relatively few microcrystalline regions in the 4REP spider protein repeat region prevent the maintenance of its stable *β*‐fold structure. With respect to the self‐assembled 6REP spidroin, the formation of nanoparticles was accompanied by long strips of aggregates. As shown in Figure 3cii, these long strips of aggregates are ≈200 nm in diameter, and the appearance of these fibrous structures indicates that 6REP spider proteins have stronger self‐assembly performance than 4REP proteins, which may be due to the increase in the density of microcrystalline regions, which increases the probability of collision between microcrystalline regions, thereby promoting the stability of *β*‐folded microcrystalline structures. Interestingly, the molecular weight of the 6REPM spider protein is similar to that of the 4REP spider protein, and the number of microcrystalline regions is the same as that of the 6rep spider protein; however, the formation of dense long strips of aggregates with the expected length of a few microns appears for the 6REP spider protein, as shown in Figure 3ciii. Combined with the results of CD analysis, these findings suggest that a more stable anti‐parallel *β*‐sheet structure may form with amyloid polypeptides at 6 Rep, and the *β*‐microcrystalline structure may further stabilize by increasing the density of the site to promote self‐assembly.

**Figure 4:**
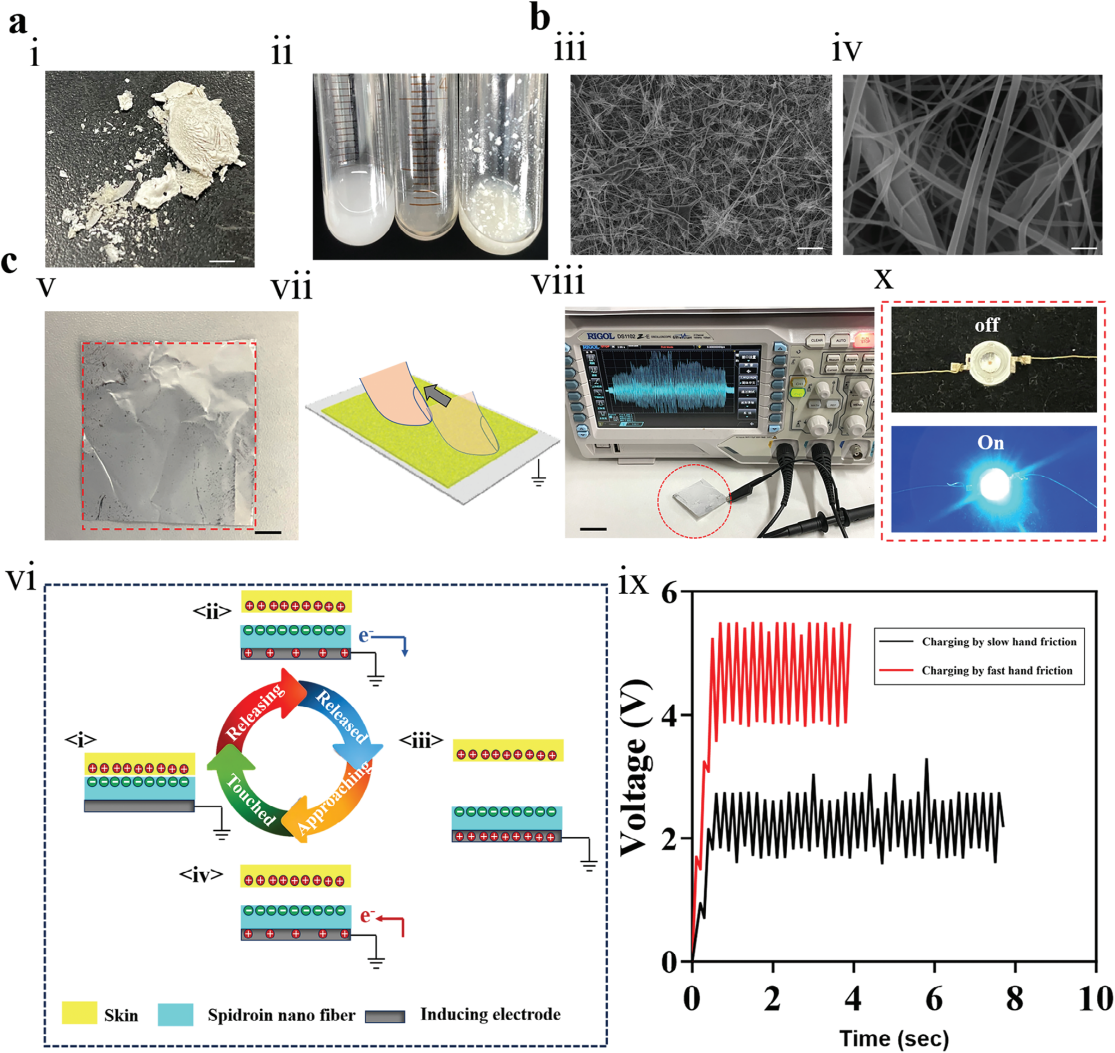
a) Solid powder of biomimetic spidroin. (i) Dissolved states of biomimetic spidroin in different solutions. From left to right, 1 mg of biomimetic spidroin is dissolved in 1 mL of pure water, 100 mg of biomimetic spidroin is dissolved in 500 µL of HFIP, and 1 mg of background *Escherichia coli* protein is dissolved in 1 mL of HFIP (ii). b) SEM image of nanospidroin fibers obtained by electrospinning (iii) and (iv). c) Physical image of a nanospidroin fiber membrane (v); schematic diagram of friction power generation of nanospidroin fiber (vi), (vii). Electric potential generated by rubbing a nanospidroin fiber membrane is recorded by an oscilloscope (viii). Charging of a 50 nF capacitor by nano spidroin fiber under slow and fast hand rubbing (ix). LEDs are lit up by electrical energy generated by rubbing nanospidroin fibers (x). Scale bar: 5 mm (i), 20 µm (iii), 2 µm (iv), 1 cm (v), 2 cm (viii).

Combined with the above experimental results, we decided to conduct spinning experiments on Amy‐6rep, which had the highest yield and the strongest self‐assembly performance. The inclusion body of Amy‐6rep was purified by washing the inclusion body and freeze‐drying (**Figure** **4**ai). The freeze‐dried protein was dissolved in HFIP solution to prepare the spinning solution. Notably, Amy‐6rep is highly soluble in HFIP, as other studies have reported,^[^ ^28^ ^]^ as shown in Figure 4aii. From left to right, 1 mg of Amy‐6rep spidroin was dissolved in pure water (insoluble), 200 mg of Amy‐6rep spidroin was dissolved in the HFIP state (light yellow transparent state), and 1 mg of substrate expression protein was dissolved in pure water (insoluble). The high solubility of Amy‐6rep in HFIP provides a good basis for the preparation of subsequent spinning solutions. As shown in Figure 4b, a nanospider fiber with a diameter of 500 nm and a uniform distribution can be prepared by electrospinning at a voltage of 18.82 kV using a high‐concentration Amy‐6rep solution without any other added ingredients. The surface morphology of Amy‐6rep was characterized by scanning electron microscopy. As shown in Figure S4 (Supporting Information), the diameter of the fibers prepared by the electrospinning solution ranged from 400 to 900 nm and was mainly distributed at 500 nm, which is similar to the fiber diameter distribution reported by Seeram Ramakrishna's team.^[^ ^29^ ^]^ These results show that the bionic spider silk protein Amy‐6rep has good electrospinning stability. On the other hand, we found that the thin film formed by the nanospidroin fibers (Figure 4cv) has good triboelectric power generation capacity. Rubbing the nano spidroin fiber film against the skin can help the surface of the film gain and lose electrons, thereby generating electrical energy, as shown in the schematic diagram (Figure 4c,vi,vii). An oscilloscope was used to record the potential generated by the friction of the nano spidroin fiber membrane (Figure 4c,viii). In the process of slow friction, a potential difference of ≈2–3 V was generated, and in the process of fast friction, the potential difference increased to 5–6 V (Figure 4c,ix), which indicated that the nanospidroin fiber membrane could provide electrical energy for the nanogenerator. Therefore, we used a customized low‐cost controllable friction device to apply constant friction to the nanospidroin fiber membrane while connecting capacitors at both ends of the friction device and a low‐power LCD bulb. The capacitor is charged under the consistent friction of the nanospidroin fiber film, which finally lights up the LCD bulb, confirming the potential of the nanospidroin fiber for use in nanogenerators. At the same time, to utilize the good biocompatibility of spider silk protein and the power generation capacity of nanofriction for effective wound management, we conducted an experimental verification of wound management in diabetic mice. The results showed that the Amy‐6rep nanofibers provided comprehensive and effective support for wound healing and promoted tissue repair by regulating cell proliferation, differentiation, and migration at the wound site in diabetic mice. Throughout the recovery phase, we observed the wound area on days 0, 3, 6, 9, and 12. As shown in Figure S5 (Supporting Information), the spider silk + friction power generation group had the most favorable wound degree. The wound healing rate was calculated using ImageJ software for further quantitative analysis (Figure S5c, Supporting Information). The experimental results clearly showed that the treatment effect of the spider fiber group on wound healing was greater than that of the control group, and the effect of the spider fiber self‐friction power generation group was greater than that of the spider fiber group. This finding confirms that the self‐friction generation of the bionic spider silk protein Amy‐6rep electrospun fiber is a more effective method for wound repair.

## 3 Conclusion 

In conclusion, we combined and further optimized the strategies previously used to improve the yield and silk‐forming performance of recombinant spidroin, achieved a balance between spinning performance and heterologous expression efficiency, generated a low‐molecular‐weight bionic spidroin (Amy‐6rep) that can be prepared in large quantities, and verified its excellent spinning performance by electrospinning. It is used in nanogenerators. Owing to the low sequence repetition of the amyloid polypeptide in bionic spidroin, the yield of homologous expression of the bionic spidroin Amy‐6rep increased by ≈200%, and the simple purification method was combined to improve the recovery efficiency, laying the foundation for subsequent mass spinning. Our study demonstrated that there is still much room for optimizing the sequence of recombinant spidroin. To further improve the heterologous expression efficiency and spinning performance of recombinant spidroin, additional studies are needed. First, we investigated the types of polypeptides with high filament‐forming properties in the amyloid polypeptide database, selected suitable amyloid polypeptides through molecular dynamics simulation combined with protein allogenic easy expression analysis, and replaced the polyalanine sequence in the microcrystalline region. In addition, we can further improve the heterologous expression efficiency of spidroins by designing flexible region sequences of spidroins and replacing characteristic motifs with high Gly contents via structurally similar sequences. Through these improvements, we believe that bionic spidroins will open up a new path for the industrial production of artificial spider silk and inspire additional inspiration for related applications.

## 4 Experiment Section

### Construction and Expression of Biomimetic Spidroins

All biomimetic spidroin sequences are shown in Figure S1 (Supporting Information). All biomimetic spidroin genes constructed from the cloning vector pUC57 were synthesized by GE Healthcare (Chicago, IL). All biomimetic spidroin genes were inserted into a 6× His‐labeled pSE380 expression vector by one‐step cloning. The three constructed plasmids were subsequently transformed into the *E. coli* strain Rosetta (DE3). The correct colony was incubated with LB medium (50 mL with 100 µg mL^−1^ ampicillin) in an orbital shaker overnight at 37 °C, after which 2% of the 50 mL culture was transferred to 100 mL of LB medium supplemented with 100 µg mL^−1^ ampicillin, after which the mixture was inoculated at 37 °C until the OD_600_ reached ≈0.8. The induction conditions were optimized as shown in Figure S1 (Supporting Information). Finally, the cell pellets were resuspended in Buffer A (20 mm Tris, 300 mm NaCl, pH 8.0) and lysed by sonication followed by centrifugation at 16 000 × g at 4 °C for 30 min. On the one hand, the cell lysate supernatant was loaded into a Ni‐NTA column and sequentially washed with Buffer B (20 mm Tris, 300 mm NaCl, 300 mm imidazole, pH 8.0) at concentrations of 10%, 20%, and 30%. For subsequent self‐assembly characterization, all three biomimetic spidroins were diluted to the same concentration. The protein concentration was determined by the BCA method. On the other hand, the precipitation of cell lysates was performed with the inclusion body washing method. Inclusion body rinsing buffer was added for resuspension [500 mmol L^−1^ NaCl, 20 mmol L^−1^ Tris‐Cl pH 8.0, 1 mol L^−1^ urea, 1% v/v Triton X‐100], and the mixture was centrifuged at a low temperature for 10 min (10 000 rpm, 4 °C); the supernatant was discarded, and the process was repeated twice. After the second wash, the rinse buffer did not contain 1% v/v Triton X‐100. After two rinses, an appropriate amount of inclusion body cracking buffer (500 mmol L^−1^ NaCl, 4 mol L^−1^ urea, and 20 mmol L^−1^ Tris‐Cl, pH 8.0) was added to resuspend the fragments. After pyrolysis (160 rpm, 2 h) and centrifugation at low temperature for 10 min (10,000 rpm, 4 °C), the supernatant was collected, dialyzed against 1% acetic acid, lyophilized, and stored at −80 °C until use.

### SDS‒PAGE and Purity Analysis

All the SDS‒PAGE gels were 1.5 mm thick, with a 5% concentrated gel at the top and a 10% separated gel at the bottom. The samples were prepared in SDS‒PAGE loading buffer (4×) (with DTT) (2% SDS, 10% glycerol, 60 mm Tris pH 6.8, 0.01% bromophenol blue, 100 µm DTT). The gel was run on PROTEAN Tetra Cells (Bio‐Rad) in 1× Tris‐glycine SDS buffer (25 mm Tris base, 250 mm glycine, 0.1% w/v SDS) until the dye front end left the separation gel. Protein expression levels were estimated by integrating the strength of the product bands with the sum of all protein bands on the gel using ImageJ. SDS‒PAGE and purity analysis were subsequently performed.

### Circular Dichroism

Circular Dichroism (CD) spectra were obtained by using a Jasco J‐1500 spectrometer at 25 °C. The self‐assembled biomimetic spidroins were diluted to 0.1 mg mL^−1^ for measurement using glass cuvettes with a path length of 1 cm. Wavelength scans were collected from 185 to 260 nm in 0.5 nm steps with a scan speed of 100 nm min^−1^ and a bandwidth of 1.0 nm.

### Fourier Transform Infrared Spectroscopy

The secondary structures of the recombinant spidroins were characterized via FTIR spectroscopy in ATR mode (Bruker Optik Gmb H Tensor II). For each measurement, the wavenumbers ranged from 400 to 4000 cm^−1^ with 128 scans and a resolution of 8 cm^−1^. A nitrogen atmosphere was used to avoid interference with water absorption. In addition, deconvolution of the amide I band was performed using PeakFit 4.04 software.

### Atomic Force Microscopy

For atomic force electron microscopy (AFM) measurements, self‐assembled recombinant spidroin dispersions were diluted to 0.1 mg mL with deionized water. Then, 10 µL of the aqueous solution was added onto a clean mica surface for coating for 120 s, followed by purging with nitrogen gas. The morphologies of the nanoparticles and nanofibrils were observed by an ICON AFM fast scanning system (Bruker ICON). A silicon tip with a nominal spring constant of 0.4 N m^−1^ (ScanAsyst‐Air, Bruker) was used for the AFM measurements. The morphological data of self‐assembled proteins were processed by the software NanoScope Analysis 1.8.

### Electrospinning of Nanofibrous Membranes

Lyophilized protein powders were dissolved in HFIP to prepare spinning dopes at a concentration of 12% w/v. The dopant was then loaded into a 1 mL syringe connected to a 30 G steel needle (Braun). The needle was loaded into the electrospinning device. The aluminum foil length was 10 cm, and the electrospinning distance was 6 cm. The cone angle was 60°, the needle length was 2 cm, and a voltage of 18.82 kV was applied for spinning at a rate of 0.6 mL h^−1^. The electrospinning temperature was maintained at 25 ± 2 °C, and the relative humidity was 50±5%.

### SEM

After electrospinning, the foil containing the nano biomimetic spidroin fiber was fixed to the sample holder with conductive tape. The sample holder was sputtered with 10 nm of gold using a Leica EM ACE600 high vacuum sputtering coator (Leica Microsystems). Optical fibers were imaged with a Nova NanoSEM 230 field emission scanning electron microscope (field electron and Ion Company, FEI) at an accelerating voltage of 10 kV.

### Test and Measurement of Nanofibrous Membranes

The diameter of the nanofibers was measured using Nano Measure 1.2 software. A custom‐built low‐cost, controlled friction device, as shown in Figure 4viii, is used to apply a constant force to a nanogenerator. The generated output voltage was captured using a digital oscilloscope (DSO 01012A, Agilent Technologies). The current of the nanofibrous membrane was evaluated using an ultralow input current amplifier, the LMC6001 operational amplifier. The counter consists of a coparameter‐based operational amplifier, a microcontroller, and an LCD display. The nanogenerator is composed of a low‐power LCD lightning product and a 50 nF capacitor charged by two nanospidroin fiber membranes under consistent rubbing.

### Bioproduction in Fed‐Batch Bioreactors

Fed‐batch fermentation of Amy‐6rep from the Pse380 vector *E. coli* BL21(DE3) strain (Vazyme Biotech Co., Ltd.) was performed. Specifically, cells were cultivated in 400 mL of TB media supplemented with 50 µg mL^−1^ ampicillin until the OD600 reached ≈5. The culture was then centrifuged, and the pellet was resuspended in 2 L of batch media (60 g L^−1^ glycerol, 20 g L^−1^ tryptone, 24 g L^−1^ yeast extract, 10 g L^−1^ glucose, 0.5 g L^−1^ magnesium sulfate heptahydrate, 3.4 g L^−1^ potassium phosphate monobasic, 3.6 g L^−1^ sodium phosphate dibasic, 2.7 g L^−1^ ammonium chloride, 0.7 g L^−1^ sodium sulfate, 100.8 mg L^−1^ ferric citrate, 2.5 mg L^−1^ cobalt chloride hexahydrate, 15 mg L^−1^ manganese chloride tetrahydrate, 1.5 mg/L copper chloride dihydrate, 3 mg/L boric acid, 2.1 mg L^−1^ sodium molybdate dihydrate, 33.8 mg L^−1^ zinc acetate hydrate, 14.1 mg L^−1^ EDTA, 50 mg L^−1^ ampicillin, 0.01% v/v antifoam 204) and transferred to a 5 L Bioflo120 fed‐batch bioreactor (Eppendorf, Hamburg, Germany). The culture was controlled at 37 °C, and the pH was maintained at 7.1 with either 3 m phosphoric acid or 25% ammonia. Autoclaved water was added to the system to compensate for water loss during fermentation. The DO level was initially set at 70% and maintained at no less than 30% by adjusting the stirring speed (200–1200 rpm) and the airflow rate. The cells were induced at OD600 ≈ 18 with a final IPTG concentration of 0.25 mm at 35 °C. Feed media (400 g L^−1^ glycerol, 20 g L^−1^ tryptone, 24 g L^−1^ yeast extract, 40 g L^−1^ magnesium sulfate heptahydrate, 3.4 g L^−1^ potassium phosphate monobasic, 3.6 g L^−1^ sodium phosphate dibasic, 2.7 g L^−1^ ammonium chloride, 0.7 g L^−1^ sodium sulfate, 40 mg L^−1^ ferric citrate, 4 mg L^−1^ cobalt chloride hexahydrate, 23.5 mg L^−1^ manganese chloride tetrahydrate, 2.3 mg L^−1^ copper chloride dihydrate, 4.7 mg L^−1^ boric acid, 4 mg L^−1^ sodium molybdate dihydrate, 16 mg L^−1^ zinc acetate hydrate, 13 mg L^−1^ EDTA, 50 mg L^−1^ ampicillin, 0.01% v/v antifoam 204) were added at a rate of 0.2 mL mi^−1^n. The pellets were collected 32 h after induction. The estimates of the protein expression levels and titers are shown in Figure S3 (Supporting Information).

### In Vitro Wound Healing Experiment

A 1 cm long round skin wound was cut on the back of the animal after anesthesia with a scalpel. The mice were randomly divided into 5 groups: the control group (untreated group), the spidroin fiber group, and the spidroin fiber+friction group. To ensure smooth skin penetration, the dressing was applied to the wound and pressed for 1 min. The experimental dressing was changed every 24 h. The wounds were photographed on days 0, 3, 6, 9, and 12 for analysis.

All volunteers in the experiments gave their full, informed, voluntary consent to participate in this research. All animal operations were approved and authorized by the Scientific Ethical Committee of the School of Pharmaceutical Sciences, Nanjing Tech University. All animal experiments were performed in strict accordance with the “Guidelines for the Care and Use of Laboratory Animals” and received approval from the Animal Investigation Ethics Committee of Nanjing Tech University (No. LL‐2022040201).

### Statistical Analysis

All the statistical data are expressed as the mean ± standard deviation, *n* = 6 for each group. One‐way ANOVA was used to calculate the p values. Differences with *p* < 0.05 and *p* < 0.01 were considered statistically significant and are labeled with ^*^ and ^**^, respectively. Origin 2022 was used for the statistical analysis.

## Conflict of Interest

The authors declare no conflict of interest.

## Supporting information

Supporting Information

## Data Availability

The data that support the findings of this study are available from the corresponding author upon reasonable request.
