# Supplementary material for: Efficient Biosynthetic Fabrication of Spidroins with High Spinning Performance
Source: Adv Sci (Weinh). 2024 Mar 23;11(22):2400128. doi: 10.1002/advs.202400128 (PMC11165546; doi:10.1002/advs.202400128)
Supplement: Supplementary file 1 — Supporting Information [file ADVS-11-2400128-s001.pdf]

ADVANCED SCIENCE
Open Access

Supporting Information

for *Adv. Sci.*, DOI 10.1002/advs.202400128

Efficient Biosynthetic Fabrication of Spidroins with High Spinning Performance

Baoyang Lin, Jingjun Xie, Bingbing Gao and Bingfang He**

Supporting Information

Efficient Biosynthetic Fabrication of Spidroins with High Spinning Performance

Baoyang Lin, Jingjun Xie, Bingbing Gao and Bingfang He**

School of Pharmaceutical Sciences, College of Biotechnology and Pharmaceutical Engineering, Nanjing Tech University, Nanjing 211816, China.

*E-mail: gaobb@njtech.edu.cn, bingfanghe@njtech.edu.cn.

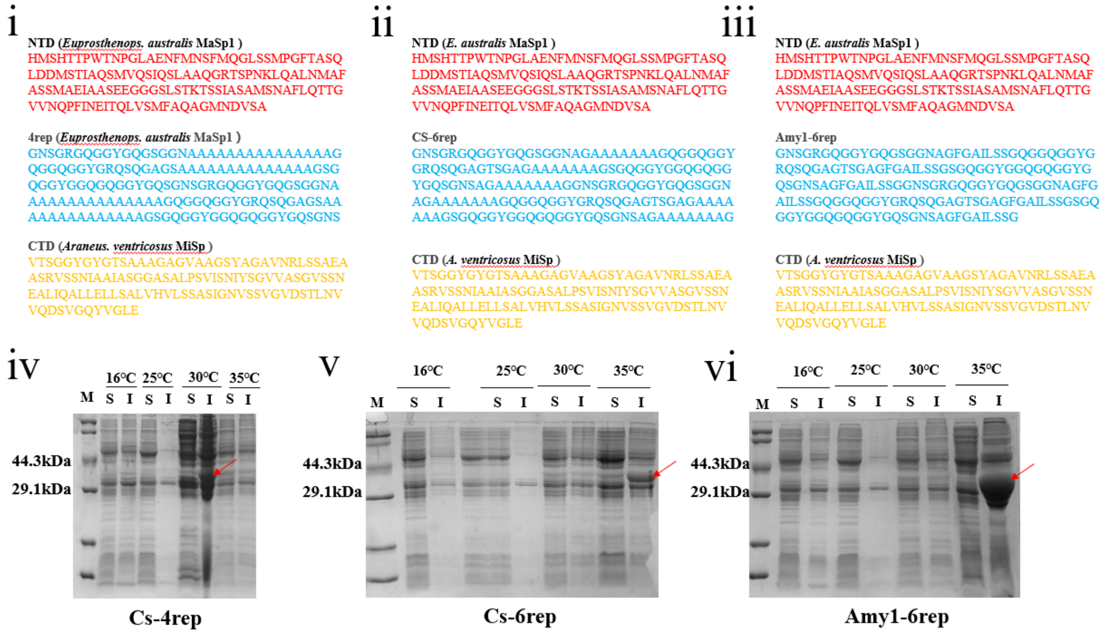

Figure S1. Optimization of the amino acid sequences of three biomimetic spidr oins. 4rep (i); Cs-6rep (ii); Amy-6rep (iii). Fermentation conditions for the thre e biomimetic spidroins. 4rep (iv); Cs-6rep (v); Amy-6rep (vi).

Table S1. Summary of the recovery, yield and productivity of all the spidroins

(inclusion body form) used in this study. All the data were obtained under shake-flask fermentation conditions.

	Recovery (%)	Yield (%)	Productivity (mg purified/g dry cell)
4rep	78.3	15 ± 5	180 ±10
Cs-6rep	82.9	13 ± 2	185 ± 5
Amy1-6rep	80.4	45 ± 10	535 ± 10

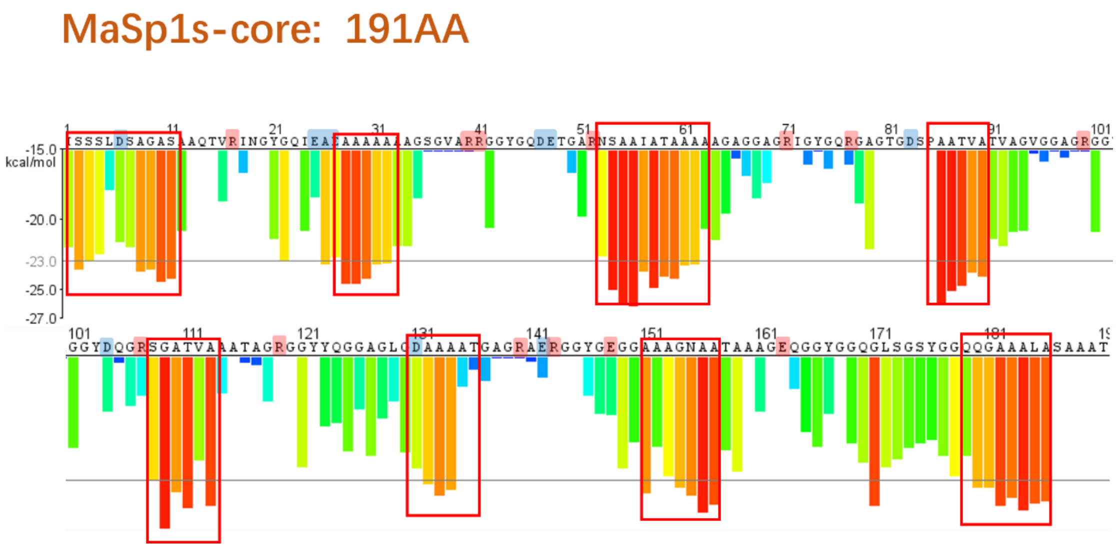

Figure S2. Rosetta energy analysis of the MaSp1s cores. The region circled by the red wire frame is the segment with strong fiber forming ability.

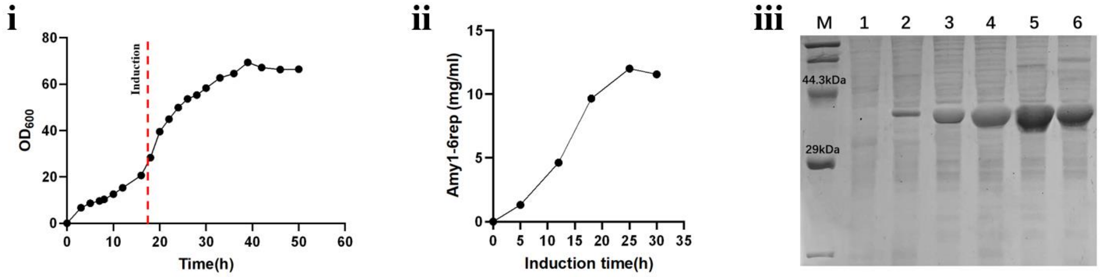

Figure S3. Expression of Amy-6rep in *E. coli* in a high-cell density culture. (i) OD600 of the *E. coli* culture over time before and after induction. The induc

tion point is indicated by the dashed line. (ii) The concentration of Amy-6rep in the culture was estimated via SDS–PAGE as a function of induction time. (iii) Amy-6rep expressed in the bioreactor was purified. M: marker. Lines: 1-6, Samples of Amy-6rep with different induction times: 1:0 h; 2:5 h; 3:12 h; 4: 18 h; 5:25 h; and 6:30 h. All the samples were diluted to an OD600 of 5 an d processed.

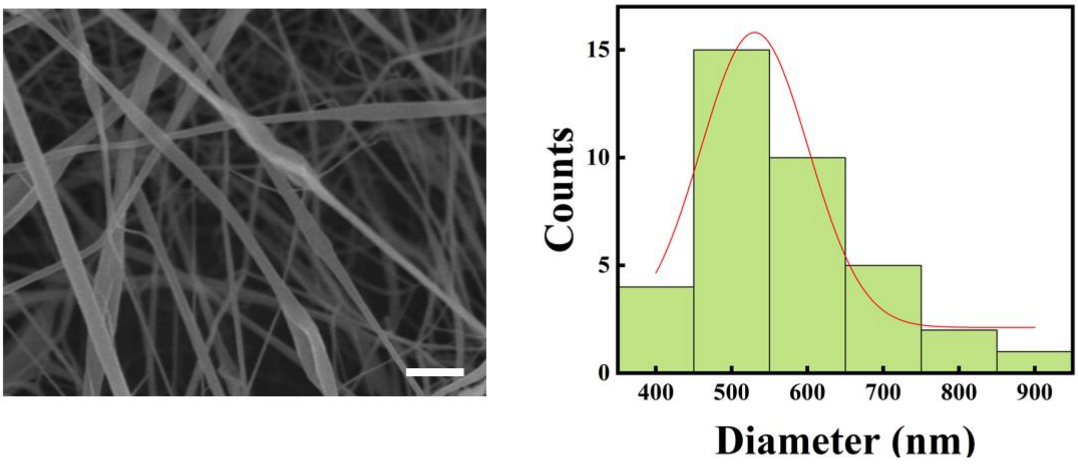

Figure S4. SEM image and fiber diameter distribution of the Amy-6rep electrospun fibers. Scale bar: 2 μm.

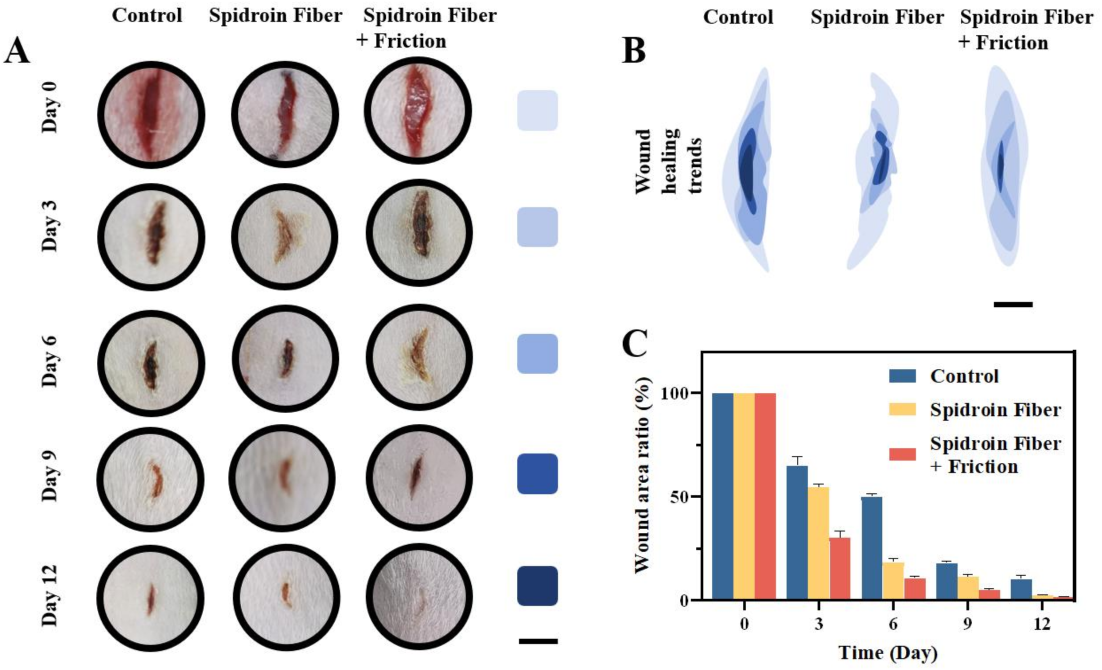

Figure S5. Evaluation of the effect of Amy-6rep nanofibers on epidermal wounds in vivo. A) Representative digital images of wounds from each group on days 0, 3, 5 and 12 (mean ±SD; n = 6) (scale bar: 6 mm). B) Contours of each group at days 0, 3, 5 and 12 (mean ±SD; n = 6) (scale bar: 6 mm). C) Quantification of the wound area (means ± SDs; n = 6).

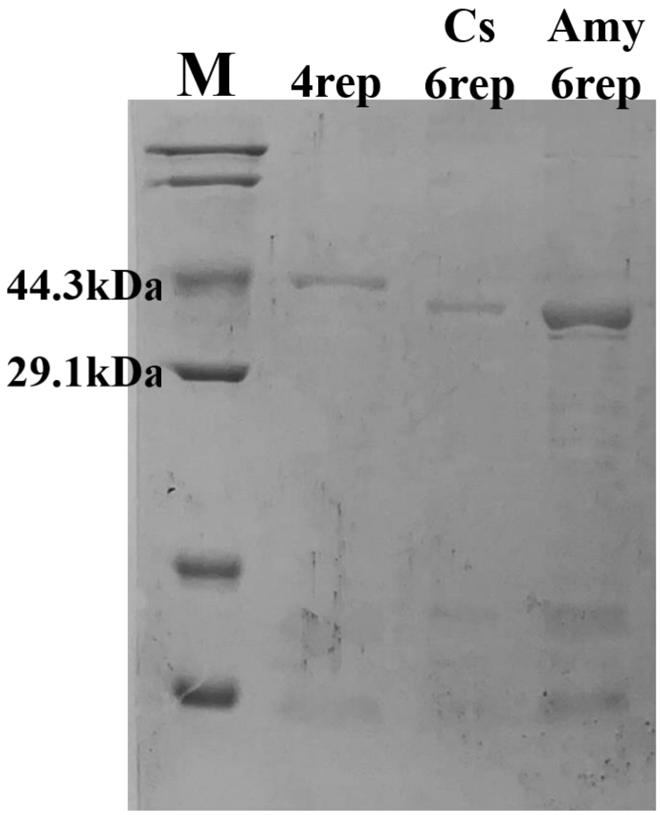

Figure S6. Ni-NTA purification of soluble parts of all bionic spidroins.
